# Colon Adenoma Implicating Myasthenia Gravis: A Case Report of a Patient with Postcolectomy Complications

**DOI:** 10.1155/2016/6297656

**Published:** 2016-08-17

**Authors:** Y. Papachatzakis, E. Tseliou, I. Tatouli, I. Dialoupi, F. Michas, E. Papadopoulou, D. Kousouris, S. Kontogiannis, M. A. Dimopoulos

**Affiliations:** Advanced Care Unit, Department of Therapeutics, Alexandra Hospital, University of Athens, Greece

## Abstract

We report the case of a 63-year-old patient with myasthenia gravis (MG) due to acetylcholine receptor antibodies (AChR) who underwent colectomy due to colon adenoma and developed myasthenic crisis and anastomosis leakage after surgery. The patient underwent two plasma exchanges, 4 and 6 days preoperatively, and received intravenous prednisolone and immunoglobulin infusion due to the crisis, which included primarily bulbar symptoms. The patient developed on the 10th postoperative day bowel obstruction symptoms and anastomosis leakage which required surgical repair and ileostomy. Bowel obstruction occurred in a patient with AChR related myasthenia after plasma exchange and during immunosuppression although it is more commonly reported in patients with thymoma related myasthenia.

## 1. Introduction

Myasthenia gravis is an autoimmune disease affecting 4–12 million people annually [1] and is caused by autoantibodies to postsynaptic muscle endplate [2]. The majority of the patients present with muscle weakness and fatigue while bulbar symptoms are present in over 80% of patients with antibodies against AChR [3]. In patients in need of surgery, preoperative plasma exchange is indicated to avoid myasthenic crisis.

## 2. Case Presentation

In October 2015, a 63-year-old man with myasthenia gravis was admitted to our Advanced Care Unit due to bulbar symptoms. The patient was diagnosed with myasthenia gravis due to AChR antibodies in 2013 and was in stable remission under pyridostigmine (30 mg t.i.d.), prednisolone (20 mg q.d.), and cyclosporine (150 mg b.i.d.). In August 2015, he was diagnosed with colon adenoma and surgical resection was indicated. Preoperatively, the patient's AChR antibody level was within normal limits (normal, <0.2 nmol/L), with no symptoms of MG apparent. The patient underwent two plasma exchange treatments with the second one being performed 4 days before surgery. On postoperative day 2, the patient experienced respiratory crisis of MG, diagnosed using the edrophonium test. The patient was supported with noninvasive positive pressure ventilation, intravenous prednisolone (25 mg twice daily), and immunoglobulin every day leading to improvement of the crisis symptoms. Plasma exchange was not performed due to respiratory infection. On the 10th postoperative day, the patient developed acutely bulbar symptoms and abdominal discomfort. Intestinal leakage was observed from the surgical site with signs of acute abdomen.

The patient was intubated and transferred to the operation room where anastomosis leakage was reported. Right colectomy and ileostomy were performed for the management of peritonitis. Intravenous prednisolone and pyridostigmine bromide were administered postoperatively due to general fatigue which was attributed to myasthenic symptoms although MG had been controlled without severe crisis symptoms. The patient progressively improved and was discharged home on day 17 after the second operation in a stable condition.

Histopathological evaluation of the resected colon revealed absence of inflammatory cell infiltrates indicative of MG related pathology. MG has since been controlled with oral cyclosporine, prednisolone, and pyridostigmine.

## 3. Discussion

Intestinal pseudoobstruction is a clinical condition characterized by the presence of bowel obstruction symptoms in the absence of any mechanical etiology [4]. It consists of a pathology commonly described in patients with MG due to the presence of concurrent thymomas [5]. Surgical thymectomy is performed for the management of the recurrent obstruction observed in these patients. Here we report for the first time the case of a patient with late-onset MG due to AChR antibodies in remission, who developed myasthenic crisis with both bulbar symptoms and bowel obstruction after colectomy for adenoma. Due to the plasmapheresis, the patient was not expected to develop a myasthenic crisis. The bulbar symptoms plus the bowel obstruction were attributed to the main disease of the patient. In such patients with AChR antibodies, gastrointestinal involvement is not commonly reported. In addition, the crisis was successfully treated with intravenous immunoglobulin. The administration of immunoglobulin instead of plasma exchange in the ACU in case of MG crisis can be equally effective to successfully treat these patients.
